# Control Efficacy of Salicylic Acid Microcapsules against Postharvest Blue Mold in Apple Fruit

**DOI:** 10.3390/molecules27228108

**Published:** 2022-11-21

**Authors:** Yifei Wang, Jiahao Chen, Wenyi Bian, Xiaobo Yang, Lin Ye, Shoukui He, Xiaoqiu Song

**Affiliations:** 1 Department of Food Science and Technology, School of Perfume and Aroma Technology, Shanghai Institute of Technology, Shanghai 201418, China; 2 Department of Food Science and Technology, School of Agriculture and Biology, Shanghai Jiao Tong University, Shanghai 200240, China

**Keywords:** β-cyclodextrin inclusion complex, *Penicillium expansum*, postharvest disease, induced resistance, fruit quality

## Abstract

Salicylic acid (SA) is a natural inducer of disease resistance in fruit, but its application in the food industry is limited due to low water solubility. Here, SA was encapsulated in β-cyclodextrin (β-CD) via the host–guest inclusion complexation method, and the efficacy of SA microcapsules (SAM) against blue mold caused by *Penicillium expansum* in postharvest apple fruit was elucidated. It was observed that SAM was the most effective in inhibiting the mycelial growth of *P. expansum* in vitro. SAM was also superior to SA for control of blue mold under in vivo conditions. Enzyme activity analysis revealed that both SA and SAM enhanced the activities of superoxide dismutase (SOD) and phenylalanine ammonia lyase (PAL) in apple fruit, whereas SAM led to higher SOD activities than SA. Total phenolic contents in the SAM group were higher than those in the SA group at the early stage of storage. SAM also improved fruit quality by retarding firmness loss and maintaining higher total soluble solids (TSS) contents. These findings indicate that microcapsules can serve as a promising formulation to load SA for increasing *P. expansum* inhibition activity and improving quality attributes in apple fruit.

## 1. Introduction

Apple is an important economic fruit in China and it contains various nutrients and bioactive compounds such as minerals, vitamins, and cellulose [1]. However, fruit is prone to fungal contamination during transportation and storage, which causes huge economic losses to the apple industry [2]. The postharvest blue mold, caused by *Penicillium expansum*, is a main factor leading to rapid decay of apple fruit [3]. Fruit wax coatings, synthetic bactericides, and other interventional treatments have been used to control this disease. However, consumers have gradually declined these intervention strategies owing to their food-safety concerns [4]. In this context, the demand for healthy, efficient, and environmentally friendly control measures for postharvest diseases in fruit is increasing.

Inducing defense responses with biological or chemical elicitors is a widely accepted approach for controlling plant diseases and stimulating long-term systemic resistance against various pathogens [5]. Salicylic acid (SA) is an endogenous phenolic hormone in willow bark, white pearl leaves, and sweet birch. It is involved in the regulation of photosynthesis, mineral absorption, and plant growth [6]. SA can trigger defensive mechanisms and promote metabolism, thereby reducing postharvest losses in fruit [7]. Some recent studies have shown that SA is effective in inhibiting *P. expansum*-induced blue mold in apple, peach, and citrus fruits [8,9,10]. However, SA is not only highly water insoluble due to its chemical structure but also presents an unpleasant smell. Therefore, SA commercialization in fruit preservation is not ideal practically and requires modern technologies such as encapsulation to expand its utility [11].

Encapsulation is an efficient technology that has been extensively used in the food, pharmaceutical, agrochemical, and cosmetics industries. Studies on the encapsulation of hydrophobic molecules have attracted increasing attention [12]. Currently, several wall materials have been developed for SA microcapsules (SAM) such as β-cyclodextrin (β-CD), chitosan, and silica [13,14]. In particular, β-CD is widely used as a wall material to encapsulate active substances with unpleasant odors, such as essential oils, owing to its advantages of inexpensiveness and non-toxicity [15]. For instance, Dou et al. [16] found that thymol/β-CD inclusion complexes reduced the sour rot of citrus fruits and maintained their quality. To the best of our knowledge, however, there is no relevant study regarding postharvest control applications of β-CD microcapsules containing SA, a natural and safe phenolic compound.

The present study aimed to determine the inhibitory effects of SAM on blue mold caused by *P. expansum* in apple fruit. The characterization of the content of antioxidant enzymes, total phenolics, and malondialdehyde was also performed to explore the possible mechanisms of the antifungal effects of SAM.

## 2. Results and Discussion

### 2.1. Effect of SAM on Control of P. expansum In Vitro

**Figure 1:**
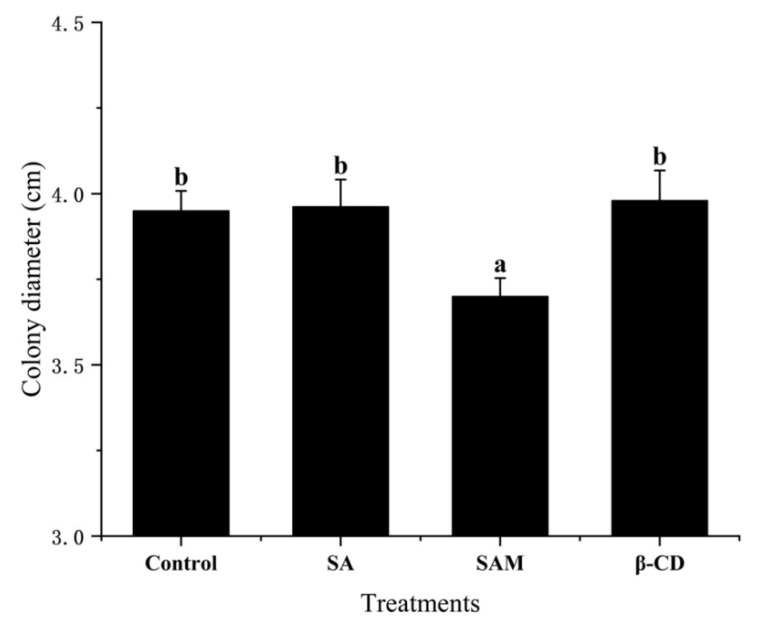
Effect of SAM on control of *Penicillium expansum* colony diameter on potato dextrose agar after 7 days of storage at 28 °C. SA, salicylic acid; SAM, salicylic acid microcapsule; β-CD, β-cyclodextrin. Control group was prepared with sterile distilled water. Vertical bars represent standard errors. Different letters show a significant difference (*p* < 0.05).

As shown in Figure 1, the mycelial growth of *P. expansum* was not significantly (*p* > 0.05) inhibited by pure SA or β-CD compared to the control group, as determined on the basis of the colony diameter. Similarly, Mandal et al. [17] found that SA at low concentrations had no direct antifungal effect on the mycelial growth of *Fusarium oxysporum* on potato dextrose agar (PDA). On the other hand, the encapsulation of SA contributed to its antifungal ability to some extent in our study (Figure 1), which was probably due to the enhanced interaction between this antifungal agent and *P. expansum* after SA was embedded. In fact, β-CD could increase the bioavailability of core materials by changing membrane permeability and protein binding [18]. Thus, SAM had the potential to serve as a feasible formulation to load SA for antifungal purposes, which needed to be checked in *in vivo* conditions.

### 2.2. Effect of SAM on Control of Blue Mold in Apple Fruit

**Figure 2:**
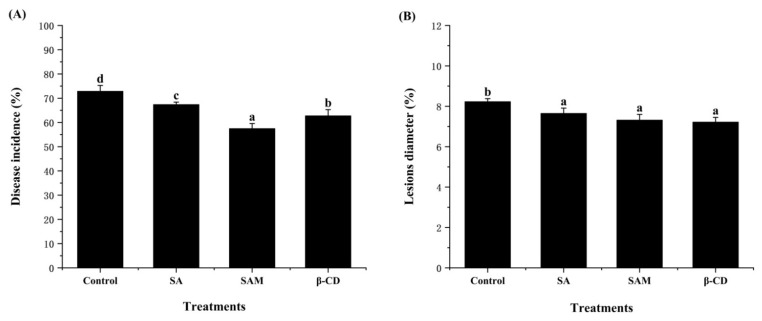
Effect of SAM on disease incidence (**A**) and lesion diameter (**B**) in ‘Fuji’ apple following *Penicillium expansum* infection after 5 days of storage at 25 °C. SA, salicylic acid; SAM, salicylic acid microcapsule; β-CD, β-cyclodextrin. Control group was prepared with sterile distilled water. Vertical bars represent standard errors. Different letters show a significant difference (*p* < 0.05).

The antifungal activity of SAM against *P. expansum* in apple fruit was also evaluated. As depicted in Figure 2, all treatments significantly (*p* < 0.05) inhibited the disease incidence and lesion diameter in apple fruit after 5 days of storage. Pure β-CD group also exerted an *in vivo* inhibitory effect against *P. expansum.* Similar research reported that β-CD could present some inhibition in the postharvest decay incidence of strawberry [19]. It was speculated that β-CD as a coating material could induce postharvest resistance in fruit [20].

In our previous work, an increased inhibition effect of clove essential oil on blue mold in orange fruit was achieved through the microencapsulation technology [21]. It is of note that the lowest disease incidence (58.1%) of blue mold was observed in the SAM group in this study (Figure 2A). This indicated that microencapsulation was also able to increase the inhibition activity of SA on blue mold in apple fruit. Considering that SA played an important role in the development of systemic resistance in apple fruit [22], it could be inferred that SAM might be more effective than SA in activating the signal transduction pathways, thus resulting in more pronounced induction of systematic resistance.

### 2.3. Effect of SAM on Antioxidant Enzyme Activities in Apple Fruit

**Figure 3:**
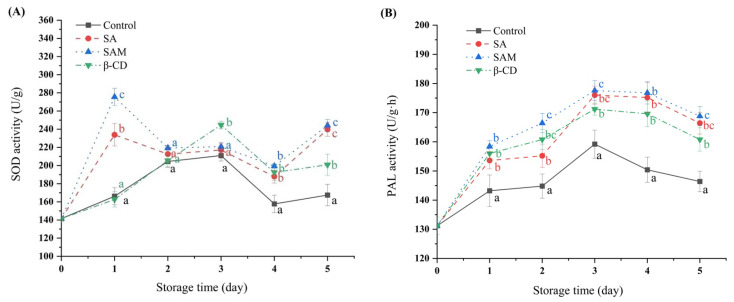
Changes in superoxide dismutase (SOD) (**A**) and phenylalanine ammonia lyase (PAL) (**B**) activities in ‘Fuji’ apple during 5 days of storage at 25 °C. SA, salicylic acid; SAM, salicylic acid microcapsule; β-CD, β-cyclodextrin. Control group was prepared with sterile distilled water. Vertical bars represent standard errors. Different letters within each day show a significant difference (*p* < 0.05).

In the present study, superoxide dismutase (SOD) activity in all treated groups increased in the initial period and then decreased (Figure 3A). SOD activity in the SA and SAM groups reached the peak on day 1, and was approximately 1.4 and 1.6 times higher than that in the control group, respectively. This also implied that the SAM group was more effective in inducing SOD activity compared with the SA group in the early stage of fungal infection. At this stage, a large amount of reactive oxygen species (ROS) is accumulated to resist pathogen invasion and further activate plant defense responses, but excessive ROS will damage plant cell-membrane lipids [23]. SOD is the main scavenging enzyme of superoxide anions, and converts superoxide anions to H_2_O_2_ and O_2_, thus reducing the damage of excessive ROS to fruits [24]. Therefore, earlier SOD activation by SAM was beneficial for relieving ROS-generated damage to apples, which subsequently slowed down their decay.

Phenylalanine ammonia lyase (PAL), a key enzyme of the phenylpropanoid pathway in plants, is involved in the synthesis of defense-response substances such as flavonoids, phenols, lignin, and SA [25]. In the current study, PAL activity in apple fruit increased at the initial stage and then decreased, and the peak value appeared on day 3 (Figure 3B). Moreover, PAL activity in the control group was lower compared with that in other groups during storage. The highest peak value of PAL activity was detected in SAM and SA groups, and was 11.6% higher than that in the control group. The superior PAL activity in these two groups could accelerate the production of some disease-resistant phenols such as total phenols and flavonoids, which were the synthetic precursors of lignin [26]. The continuous accumulation of total phenols and flavonoids during fruit infection could effectively inhibit the spread of pathogen diseases [27].

Based on the above-mentioned results, both SA and SAM induced the accumulation of antioxidant enzymes and defense-related enzymes. Furthermore, SAM-treated apple fruit had higher SOD activity and similar PAL activity compared with the pure SA-treated group. Thus, the ability of SAM to activate protective enzymes might be a reason for its pronounced inhibitory effect on blue mold in apple fruit.

### 2.4. Effect of SAM on Total Phenolic Contents in Apple Fruit

**Figure 4:**
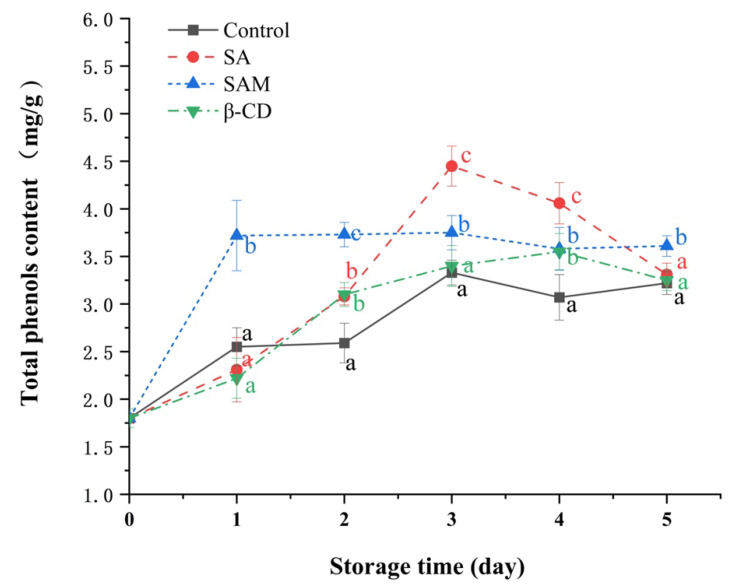
Effect of SAM on total phenolic contents in ‘Fuji’ apple during 5 days of storage at 25 °C. SA, salicylic acid; SAM, salicylic acid microcapsule; β-CD, β-cyclodextrin. Control group was prepared with sterile distilled water. Vertical bars represent standard errors. Different letters within each day show a significant difference (*p* < 0.05).

Phenols participate in the formation of secondary disease-resistance substances, including phytoprotegerin, lignin, and phenolic compounds, and are thus related to the defense responses of fruits. Some studies have suggested that the first step of plant defenses against pathogen infection is to induce the production of phenolic compounds [28]. As illustrated in Figure 4, total phenolic contents in the control and β-CD groups fluctuated and gradually increased during the whole storage period, whereas those in the SA and SAM groups firstly increased and then decreased in the later storage period. Interestingly, the peak time of total phenolic contents in different groups was different. The SAM and SA groups exhibited their peak on day 1 and day 3, respectively. In particular, total phenolic content in the SAM group was approximately 61% higher than that in the control and SA groups on the first day.

Chemical elicitors such as SA generally require a sufficient timescale to induce disease resistance in postharvest fruit [29]. In our study, although both SA and SAM groups could induce an accumulation of total phenolic contents in apple fruit, SAM stimulated this response more quickly. It was thus indicative that SAM shortened the excitation time required to induce the production of phenolics. This might because encapsulation expanded the contact area between SA and the fruit surface [30].

### 2.5. Influence of SAM on Malondialdehyde Contents in Apple Fruit

**Figure 5:**
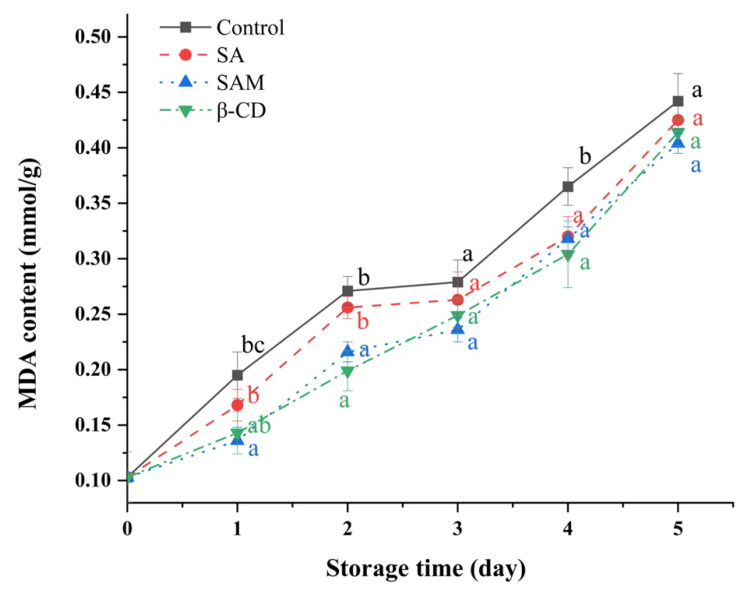
Effect of SAM on malondialdehyde (MDA) contents in ‘Fuji’ apple stored at 25 °C for 5 days. SA, salicylic acid; SAM, salicylic acid microcapsule; β-CD, β-cyclodextrin. Control group was prepared with sterile distilled water. Vertical bars represent standard errors. Different letters within each day show a significant difference (*p* < 0.05).

The content of malondialdehyde (MDA), the final product of lipid peroxidation, has been used as a direct index of cell oxidative damage [31]. When the fruit is senescent and seriously damaged, MDA content increases [32]. As shown in Figure 5, MDA content in all fruit samples increased during the whole storage period. Meanwhile, MDA accumulation in apple fruit was inhibited to some extent by both SA and SAM in comparison to that in the control group. This could be due to the stimulation of SOD activity, which led to ROS elimination and subsequent reduction in oxidative damage [33]. Similarly, Mo et al. found that SA was able to decrease the MDA content of sugar apple fruit [34].

### 2.6. Impact of SAM on Postharvest Quality of Apple Fruit

SA has been reported to maintain quality variables such as firmness for the apple fruit ‘Golden Delicious’ [35]. Firmness, the main feature of apple texture, plays an important role in consumer acceptance and shelf-life stability [36]. However, fruit firmness loss can occur during postharvest storage as a result of pectin breakdown [37]. In this study, the firmness of treated samples was significantly (*p* < 0.05) higher than that of the control ones for most of the time points (Table 1). At the end of storage, SAM exhibited the best ability to maintain the firmness of apple fruit compared with other agents. This finding suggests that SAM may contribute to the stability of pectin in apple fruit, thus maintaining higher firmness.

**Figure 6:**
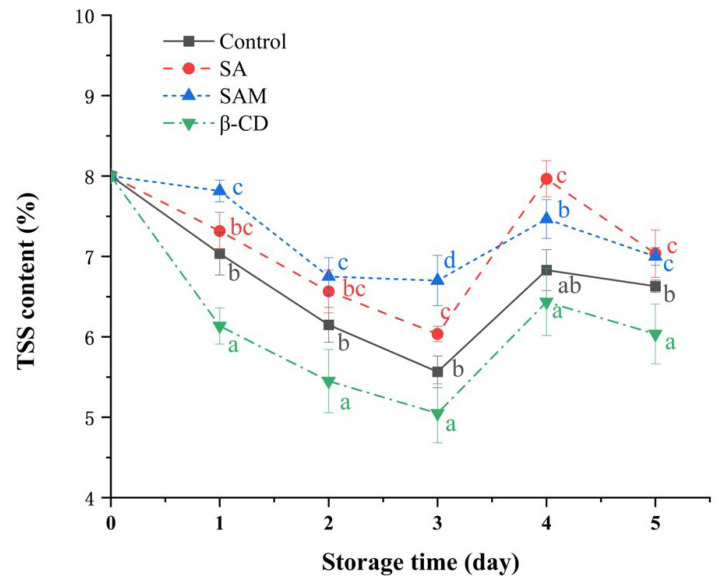
Effect of SAM on total soluble solid (TSS) content in ‘Fuji’ apple during 5 days of storage at 25 °C. SA, salicylic acid; SAM, salicylic acid microcapsule; β-CD, β-cyclodextrin. Control group was prepared with sterile distilled water. Vertical bars represent standard errors. Different letters within each day show a significant difference (*p* < 0.05).

Total soluble solid (TSS), which comprises soluble sugars such as glucose and sucrose, is considered a key substrate in an apple’s respiratory metabolism [38]. As shown in Figure 6, the TSS content in all samples continuously decreased in the early stage and then increased slightly in the subsequent period. TSS content in the SA and SAM groups was higher than that in the control group for most of the time points. This finding suggested that SA and SAM retarded the respiratory rate and sugar consumption in apple fruit. Based on our results of firmness and TSS, it could be concluded that SAM treatment was effective in maintaining the quality attributes of apple fruit.

## 3. Materials and Methods

### 3.1. Fruit Materials

‘Fuji’ apples (*Malus domestica* Borkh cv. Fuji) were harvested with a commercial level of maturity (the optimum maturity for fresh apple storage or market) from an orchard in the autumn of 2018 (Yantai City, Shandong, China) and immediately transported for laboratory analysis. Selected samples had no mechanical injury and a uniform size and firmness. Prior to each test, fruit samples were washed in 0.02% sodium hypochlorite solution, rinsed with tap water, and air-dried at 25 °C for 24 h.

### 3.2. Fungal Pathogens

The target organism *P. expansum*, isolated from decayed apple fruit, was kindly provided by Zhejiang University [39]. This strain was incubated on PDA at 25 °C for 7 days. Fungal spores were subsequently dislodged from the agar surface with sterile distilled water and adjusted to a concentration of 3 × 10^4^ spores/mL using a hemocytometer.

### 3.3. Preparation of SAM

SA was encapsulated in β-CD inclusion complexes based on the host–guest inclusion complexation method detailed in our previous work [40]. The loading capacity of SA in the resulting SAM was 30%. Our preliminary test showed that SAM at a low concentration of 165 mg/L was effective in inhibiting blue mold decay in apple fruit. This level of SAM was thus utilized in the subsequent assays, which was equivalent to 50 mg/L pure SA and 115 mg/L pure β-CD as determined via the loading capacity.

### 3.4. Effect of SAM on Control of P. expansum In Vitro

SA, β-CD, and SAM were added to liquid PDA to attain a concentration of 50, 115, and 165 mg/L, respectively. After the medium was cooled and solidified, each plate was inoculated with 10 µL of *P. expansum* suspension (3 × 10^4^ spores/mL) and incubated at 28 °C for 7 days. Colony diameters were then measured both horizontally and vertically via a cross-measurement method [41]. The average value was determined as the colony diameter (cm). Three replicates were maintained per treatment, and the experiment was performed twice.

### 3.5. Effect of SAM on Control of P. expansum In Vivo

Each apple fruit was wounded (3 mm deep × 5 mm wide) at the equator with a sterile borer and inoculated with 30 μL of the following agents: (1) 165 mg/L SAM; (2) 50 mg/L SA; (3) 115 mg/L β-CD. Treatment with sterile distilled water served as a control. After treatment for 24 h, the wounds were inoculated with 30 µL of *P. expansum* suspensions (3 × 10^4^ spores/mL). After air drying, the inoculated samples were stored at 25 °C and 80% relative humidity in trays covered with plastic films. The number of infected fruits and their lesion diameters were examined daily. Disease incidence was recorded as the number of decayed fruits/the total number of fruits. Three replicates were maintained per treatment with 12 fruits per replicate, and the experiment was performed twice.

### 3.6. Effect of SAM on Enzyme Activities and Total Phenolic Contents in Apple Fruit

Apple tissues were detached around the equator of each wound with a steel corkborer. Samples (approximately 3 g) were then mixed and immediately packed in aluminum foil, frozen in liquid nitrogen, and kept at −80 °C until use for biochemical analyses. Enzyme activities were determined using the spectrophotometer (Shimadzu, Kyoto, Japan). Three replicates were maintained with different incubation times. PAL activities were measured at 290 nm according to the methods of Mirshekari et al. [42]. SOD activity was determined at 560 nm using the method described by Wen et al. [33] and expressed as units per gram. The enzyme volume corresponding to 50% inhibition of NBT reduction was considered one enzyme unit [43]. Total phenolic contents were determined using the Folin–Ciocalteu reagent as previously described [27]. The total phenolic content of each sample was expressed as milligram gallic acid equivalent per gram (mg/g).

### 3.7. Influence of SAM on MDA Contents in Apple Fruit

MDA content (mmol/g) in apple fruit was measured according to the thiobarbituric acid (TBA) method with some modifications [44]. Samples (approximately 1 g) were ground with 5 mL of 5% (*w/v*) trichloroacetic acid in a mortar and centrifuged at 10,000 × *g* for 20 min. Afterwards, 1 mL of the collected supernatant was mixed with 4 mL of 0.67% (*w/v*) TBA, heated at 100 °C for 30 min, and then immediately cooled with ice. After centrifugation at 10,000 × *g* for 10 min, the absorbance of the supernatant was measured at 450, 532, and 600 nm and recorded as A_450_, A_532_, and A_600_, respectively. MDA concentration (mmol/g) was calculated according to the formula stated below:

MDA concentration (mmol/g) = (6.452 × (A_532_ − A_600_) − 0.559 × A_450_) (1)

### 3.8. Impact of SAM on Quality Parameters of Apple Fruit

The firmness of the apple fruit was measured using an FHM-5 hardness tester. The content of TSS in apple juice extracted from the treatment and control groups was measured using a soluble solids meter (**^o^**Brix) (Atago, Tokyo, Japan).

### 3.9. Statistical Analysis

The data were analyzed through analysis of variance in a statistical program (SPSS/PC ver. II.x, SPSS Inc. Chicago, IL, USA). Statistical significance was assessed at the *p* < 0.05 level according to a Duncan’s multiple range test [45].

## 4. Conclusions

Encapsulation technology was able to improve the inhibition efficacy of SA on the growth of *P. expansum in vitro*. Furthermore, this work also highlighted the enhanced efficacy of SAM against blue mold on postharvest apple fruit during room-temperature storage. The enhanced disease-control efficacy was potentially achieved by eliciting host defense responses through higher SOD activity and maintaining total phenolic contents. Therefore, encapsulated SA in β-CD inclusion complexes can serve as a promising agent to control postharvest loss of apples, thus contributing to the global efforts to reduce the use of fungicides and organic solvents in the fruit industry.

## Figures and Tables

**Table 1:** Effect of SAM on ‘Fuji’ apple firmness during 5 days of storage at 25 °C.

Storage Day	Firmness (N)			
	Control	SA	SAM	β-CD
0	2.82 ± 0.03 a	2.82 ± 0.03 a	2.82 ± 0.03 a	2.82 ± 0.03a
1	2.69 ± 0.20 a	2.78 ± 0.05 a	2.80 ± 0.18 b	2.80 ± 0.06 a
2	2.53 ± 0.13 a	2.76 ± 0.09 b	2.79 ± 0.13 b	2.62 ± 0.09 a
3	2.68 ± 0.13 a	2.84 ± 0.15 b	2.72±0.06 ab	2.72±0.16 ab
4	2.58 ± 0.08 a	2.73 ± 0.15 b	2.93 ± 0.05 c	2.71 ± 0.15 b
5	2.55 ± 0.11 a	2.58 ± 0.10 a	2.77 ± 0.04 c	2.67 ± 0.10 b

Note: Different letters in a low indicate a significant difference according to Duncan’s multiple range test at *p* < 0.05. SA, salicylic acid; SAM, salicylic acid microcapsule; β-CD, β-cyclodextrin. Control group was prepared with sterile distilled water. Data are expressed as means ± standard errors.

## Data Availability

Not applicable.

## Sample Availability

Not available.
